# Chlorinated Didemnins from the Tunicate *Trididemnum solidum*

**DOI:** 10.3390/md11114478

**Published:** 2013-11-11

**Authors:** Sridevi Ankisetty, Shabana I. Khan, Bharathi Avula, Deborah Gochfeld, Ikhlas A. Khan, Marc Slattery

**Affiliations:** 1 Department of Pharmacognosy, School of Pharmacy, The University of Mississippi, University, MS 38677, USA; E-Mails: skhan@olemiss.edu (S.I.K.); ikhan@olemiss.edu (I.A.K.); 2 National Center for Natural Products Research, Research Institute of Pharmaceutical Sciences, The University of Mississippi, University, MS 38677, USA; E-Mails: bavula@olemiss.edu (B.A.); gochfeld@olemiss.edu (D.G.)

**Keywords:** chlorinated didemnins, anti-inflammatory activity, *Trididemnum solidum*

## Abstract

Chemical investigation of the tunicate *Trididemnum solidum* resulted in the isolation of two new chlorinated compounds belonging to the didemnin class, along with two known compounds didemnin A and didemnin B. The structural determination of the compounds was based on extensive NMR and mass spectroscopic analysis. The isolated compounds **1**–**4** were tested for their anti-inflammatory activity using *in vitro* assays for inhibition of inducible nitric oxide synthase (iNOS) and nuclear factor-kappa B (NF-κB) activity. The anti-cell proliferative activity of the above compounds was also evaluated against four solid tumor cell lines.

## 1. Introduction

Didemnins belonging to the class of depsipeptides were first isolated from the Caribbean tunicate *Trididemnum solidum* (Family Didemnidae) [1,2]. Didemnin B, a potent anticancer agent was the first marine natural product that entered into phase I clinical trials. Didemnin B exhibited a variety of biological activities which includes inhibition of the growth of both RNA and DNA viruses [3], *in vitro* and *in vivo* activity against B16 melanoma, *in vivo* activity against P388 leukemia and *in vitro* activity against L1210 lymphocytic leukemia [4,5]. In the course of our search to identify new drug candidates and targets, the tunicate *Trididemnum solidum* (KY10508001) was collected from the Little Cayman Island and the freeze dried sample was extracted with CH_2_Cl_2_/MeOH (1:1 v/v). The extract was found to exhibit strong anti-inflammatory activity using *in vitro* assays for inhibition of inducible nitric oxide synthase (iNOS) and nuclear factor-kappa B (NF-κB) activity. The IC_50_ values were 0.2 µg/mL and 0.4 µg/mL for inhibition of iNOS and NF-κB, respectively. The crude extract was further subjected to a series of chromatographic separations to yield the didemnin class of pure compounds (**1**–**4**), and the isolated compounds were tested for their *in vitro* anti-inflammatory and anticancer activities.

## 2. Results and Discussion

### 2.1. Bioassay-Guided Isolation

**Figure 1:**
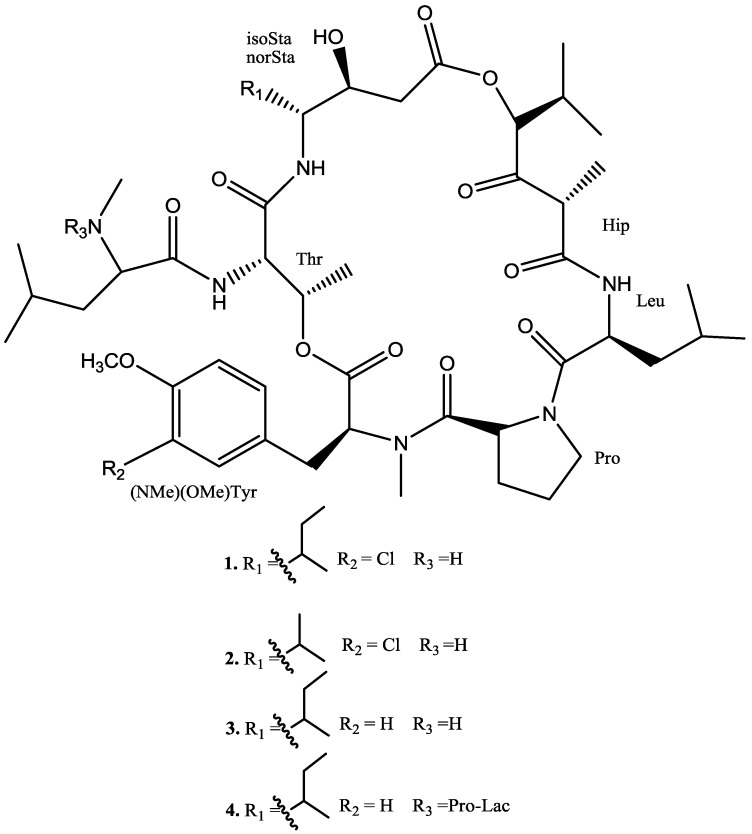
Structure of compounds **1**–**4**.

The crude DCM extract of the tunicate (5.5 g) was subjected to C_18_ flash column chromatography using water and methanol mixtures. Based on the anti-inflammatory activity, fraction D (IC_50_ of 0.14 µg/mL and 0.028 µg/mL for NF-κB and iNOS respectively) was further subjected to reversed phase HPLC (Phenomenex, Luna C_18_ (2)), using a gradient mixture (60:40 MeOH: H_2_O to 100% MeOH with 0.05% TFA over 65 min) to afford four pure compounds (**1**–**4**) (Figure 1).

### 2.2. Structural Elucidation of the New Compounds

Compound **1** was isolated as white solid with molecular formula of C_49_H_77_ClN_6_O_12_ as deduced by HRESIMS *m/z* 977.53505/979.53655 (actual 977.53662) corresponding to [M + H]^+^ ion. 49 resonances were observed in the ^13^C NMR spectrum (Supplementary Figure S1), which were identified to be 11× C (of which 8 were carbonyls); 17× CH; 8× CH_2_; 13× CH_3_ based on DEPT spectral data. Analysis of the ^1^H NMR data (Supplementary Figure S2) revealed the structural similarities of compound **1** to didemnin A [6]. Mass spectral data (Supplementary Figure S3) in combination with the ^1^H NMR spectrum (Table 1) indicated the presence of *N*,*O*-diMe-*o*-chlorotyrosine with signals at δ 7.02 (d), 112.7, 7.26 (d), 129.3, 7.43 (s), 131.5. HMBC correlations were observed from H-8 to C-6 & C-4; H-9 to C-7 & C-5; H-5 to C-7& C-9 indicating the position of chlorine to be ortho to the methoxy group on the tyrosine moiety. The structure of chlorinated tyrosine moiety was also confirmed by comparison of the data to the literature [7,8]. Analysis of the 1D (^1^H, ^13^C, DEPT) and 2D (COSY, HMQC, HMBC and ROESY) identified the rest of the amino acids as proline, leucine, threonine and Me-leucine. The stereochemistries of all the standard amino acids except for *N*-Me-d-leucine were determined to be l by LC-MS analysis (Supplementary Figure S4) of Marfey’s derivatives [9]. The stereochemistry of chloro *N*,*O*-di Me Tyr was determined by synthesis of the same using *N*,*O*-di Me-l-Tyr and sulfuryl chloride [10]. Comparison of spectral data of **1** with the literature [11,12] revealed the stereochemistry of isoSta and Hip moieties to be similar to that in didemninA. Thus, the structure of compound **1** was established as *N*,*O*-diMe-*o*-chlorotyrosine derivative of didemnin A.

**Table 1:** ^13^C NMR and ^1^H NMR data of compound **1** & **2** in *d_5_*-pyridine.

		1 ^a^		2 ^b^	
Subunit	C_#_	δ_C_	δ_H_ (*J* in Hz)	δ_C_	δ_H_ (*J* in Hz)
IsoSta or norSta	NH	-	7.62 (d, 9.5)	-	7.8 (d, 9.8)
	CO	169.1	-	169.7	-
	C_2_H_2_	40.7	2.9 (m)/4.06 (d, 17.0)	40.8	3.02 (m)/4.03 (d, 18.0)
	C_3_H	67.1	4.68 (t, 10)	67.6	4.69 (t, 10.2)
	C_4_H	56.3	4.66 (brt, 9.5)	57.7	4.51 (brt, 9.7)
	C_5_H	34.5	2.51 (m)	27.7	2.5 (m)
	C_6_H_2_/C_6_H_3_	28.1	1.45/1.73 (m)	17.2	1.16 (d, 6.6)
	C_7_H_3_	12.1	1.07 (t, 7.5)	-	-
	C_5_CH_3_	14.6	1.18 (d, 7.0)	22.1	1.22 (d, 6.3)
Hip	CO	172.2	-	172.5	-
	C_2_H	49.4	4.27 *	49.9	4.34 (q, 6.9)
	C_3_	204.6	-	208.8	-
	C_4_H	80.2	5.63 (d, 4.0)	80.4	5.61 (d, 4.0)
	C_5_H	29.6	2.39 m	29.8	2.43 (m)
	C_6_H_3_	18.9	0.86 (d, 6.0)	19.2	0.83 *
	C_5_CH_3_	16.9	0.84 (d, 6.0)	17.1	0.85 *
	C_2_CH_3_	14.8	1.49 (d, 7.0)	15.2	1.53 (d, 6.7)
Leu	NH	-	8.31 (d, 9.0)	-	8.35 (d, 9.0)
	CO	171.2	-	171.4	-
	C_α_H	49.4	5.21 (brt, 9.5)	49.8	5.22 (brt, 9.4)
	C_β_H_2_	41.8	1.96/1.68 (m)	41.9	1.79/1.48 (m)
	C_γ_H	24.7	1.70 (m) *	24.9	1.9 (m)
	C_δ_H_3_	20.9	0.83 (d, 6.0)	21.3	0.94 *
	C_δ′_H_3_	22.0	1.03 (d, 6.0)	21.5	1.03 *
Pro	CO	170.6	-	169.3	-
	C_α_H	57.8	4.83 (m)	58.8	4.83 (m)
	C_β_H_2_	27.7	1.96 (m),1.71 (m)	27.9	2.04 (m), 1.78 (m)
	C_γ_H_2_	24.8	1.63 (m), 1.91 (m)	24.9	1.58 (m), 1.90 (m)
	C_δ_H_2_	47.3	3.50 (m), 3.60 (m)	47.5	3.50 (m), 3.58 (m)
Cl-*N*,*O*-Me_2_Tyr	CO	170.6	-	170.9	-
	NCH_3_	38.4	2.67 (s)	38.8	2.66 (s)
	C_α_H	65.3	4.28 *	65.7	4.26 (dd, 9.5, 5.2)
	C_β_H_2_	33.9	3.50 (m)	33.9	3.55 (m)
	C_γ_	131.6	-	131.2	-
	C_σ_H	131.5	7.43 (s)	131.8	7.44 (s)
	C_ε_	122.1	-	122.4	-
	C_ξ_	154.1	-	154.5	-
	C_ρ_H	112.7	7.02 (d, 8.0)	113.2	7.02 (d, 8.0)
	C_ψ_H	129.3	7.26 (d, 8.0)	129.7	7.28 (d, 8.0)
	OCH_3_	55.9	3.78 (s)	56.3	3.80 (s)
Thr	CO	168.9	-	169.3	-
	NH	-	10.3 (d, 6.0)	-	10.3 (d, 6.1)
	C_α_H	57.8	5.37 (brd, 6.5)	58.1	5.38 (brd, 6.5)
	C_β_H	70.4	5.74 (brd, 5.5)	70.5	5.77 (brd, 6.3)
	C_β_-CH_3_	15.9	1.64 (d, 6.0)	16.2	1.69 (d, 6.0)
MeLeu	CO	169.6	-	170.6	-
	NCH_3_	31.2	3.14 (s)	31.8	3.15(s)
	C_α_H	59.6	4.8 *	59.1	4.84 (m)
	C_β_H_2_	39.5	2.15 (m)	39.5	2.6 (m)
	C_γ_H	25.0	1.68 (m)	25.2	1.8 (m)
	C_δ_H_3_	23.4	0.84 (d, 6.0)	23.7	0.94 (d, 5.6)
	C_δ__′_H_3_	21.8	0.92 (d, 6.0)	22.9	1.03 *

^a^ 500 MHz for ^1^H NMR and 125 MHz for ^13^C NMR; ^b^ 600 MHz for ^1^H NMR and 150 MHz for ^13^C NMR; * Multiplicity assignment not possible due to overlapping signals.

Compound **2** was also isolated as a white solid and had a molecular formula of C_48_H_76_ClN_6_O_12_ deduced by HERSIMS (Supplementary Figure S5) at *m/z* 963.52073/965.52101 (actual, 963.52097) corresponding [M + H]^+^ ion. Analysis of ^1^H and ^13^C NMR data revealed structural features similar to compound **1**. The difference in mass by 14 amu’s compared to compound **1** indicated that compound **2** differed from compound **1** by a –CH_2_ unit. The absence of triplet methyl group (C_7_H_3_) from isoSta unit and the presence of a methyl doublet at δ_H_ 1.16 d indicated that the ethyl group in compound **1** was replaced by a methyl group. The stereochemistries of all the amino acids were same as in compound **1**. Several nordidemnins were reported previously [11] and analysis of 2D NMR data indicated the presence of norStatine unit in compound **2**. Thus compound **2** was identified as nor-*N*,*O*-diMe-*o*-chlorotyrosine derivative of didemnin A.

Compounds **3** & **4** were isolated as white solids and identified as didemnin A and didemnin B by comparison of spectral data to the literature [11,12,13].

The isolated compounds **1**–**4** were tested in a series of *in vitro* cell-based assays for their effects on selected targets involved in the process of inflammation and cancer. The results are presented in Table 2. Inflammation and oxidative stress are known to be linked to the development of many disorders such as cancer, organ damage, and neurodegenerative conditions. The NF-κB family of transcription factors plays a key role in inflammation, cell cycle regulation, apoptosis, and oncogenesis by controlling gene network expression [14,15]. The activation of NF-κB involves many cellular processes leading to inflammation and development of cancer [16,17]. In the assay for NF-κB activity, a luciferase construct with binding sites for specificity protein (SP-1) was used as a control because this transcription factor is relatively unresponsive to inflammatory mediators such as phorbol myristate acetate (PMA). Inducible nitric oxide synthase (iNOS) plays a key role in regulation of blood pressure, the immune system, infection, and inflammation [18]. Overproduction of nitric oxide (NO) by iNOS has been implicated in various pathological processes such as septic shock, inflammation, rheumatoid arthritis, cancer, and tissue damage [19,20]. The increase in NO response caused mainly by endotoxins and proinflammatory mediators such as lipopolysaccharide (LPS) can be reduced by anti-inflammatory agents acting as iNOS inhibitors. The isolated metabolites were also evaluated for their cytotoxicity towards four human solid tumor cell lines (SK-MEL: melanoma; KB: epidermal carcinoma; BT549: breast carcinoma and SK-OV-3: ovarian carcinoma) and non-cancer kidney cells (Vero: monkey kidney fibroblasts). The IC_50_ values for cell growth inhibition of compounds (**1**–**4**) are presented in Table 3.

**Table 2:** Inhibition of Inducible Nitric Oxide Synthase (iNOS) and Nuclear Factor-Kappa B (NF-κB) activities by compounds (**1**–**4**) *.

Compound	iNOS	NF-κB
**1**	0.4	0.26
**2**	0.42	0.62
**3**	0.19	0.19
**4**	0.002	0.03
Parthenolide ^α^	2.8	2.8

* IC_50_ values expressed in µM; ^α^ positive control.

**Table 3:** Anti-cell proliferative activity of isolated compounds (**1**–**4**) *.

Compound	SK-MEL	KB	BT-549	SK-OV-3	VERO
**1**	0.12	0.26	0.16	0.26	4.8
**2**	0.06	0.42	0.16	0.38	2.08
**3**	0.055	0.16	0.07	0.16	4.78
**4**	0.022	0.09	0.02	0.1	0.15
Doxorubicin ^α^	1.1	1.66	1.01	1.66	14

* IC_50_ values expressed in µM; ^α^ positive control.

All the compounds (**1**–**4**) showed strong activity against iNOS and NF-κB in LPS induced macrophages and PMA induced chondrocytes, respectively. The inhibition of NF-κB was not associated with the inhibition of SP-1 indicating that inhibition of NF-κB mediated transcription by these compounds was specific. Compound **4** was the most potent in inhibiting iNOS activity compared to **1**–**3**, but its effect could be related to strong cytotoxicity towards all cell lines. All four compounds strongly inhibited cell proliferation of all four cancer cell lines in comparison to doxorubicin as the control drug (Table 3). Similar to the effect of doxorubicin, the compounds (**1**–**3**) were less toxic towards noncancerous cells (Vero cells) which were included for comparison. The strong anticancer activity can be partly explained due to a strong inhibition of NF-κB mediated transcription that may cause enhanced apoptosis and cell death [21].

### 2.3. Acid Hydrolysis and Marfey Analysis

Hydrolysis of **1** & **2** was achieved by dissolving 0.4 mg of each compound in 200 µL of 6 N HCl in a round bottom flask and stirred for 18 h on sand bath at 120 °C. The sample was dried and 1 N NaHCO_3_ (200 µL) and *N*-(3-fluoro-4,6-dinitrophyenyl)-l-alaninamide [FDAA, 50 µL (10 mg/mL solution in acetone)] were added and the mixture heated at 80 °C for 10 min. The reaction mixture was cooled to room temperature and neutralized with 2 N HCl (100 µL) and diluted with 1:1 CH_3_CN:H_2_O (300 µL). 10 µL of the FDAA derivative was analyzed by LCMS using a C_18_(2) column (Luna, 5 µm, 4.6 × 150 mm) and gradient (30:70 CH_3_CN: 20 mM ammonium formate both containing 0.1% formic acid to 70:30 over 25 min). The peaks were identified by injecting dl-mixture of Marfey’s derivatives of standard amino acids except for *N*,*O*-dimethyl tyrosine where only l amino acid was used. Retention times (min) for the amino acids were as follows: l-Leu (13.5), *N*-Me-d-Leu (15.1), l-Thr (6.27), l-Pro (9.9), *o*-Cl-*N*,*O*-dimethyl l-(Tyr) (14.1).

## 3. Experimental Section

### 3.1. General

Optical rotations were measured using a JASCO DIP-370 digital polarimeter. UV spectra were recorded on a Hewlett-Packard 8452A diode array spectrometer. NMR spectra were measured on Bruker Avance DRX-500 and Varian 600MHz spectrometer. ^1^H and ^13^C NMR spectra were measured and reported in ppm using *d_5_*-pyridine solvent peak (δ_H_ 8.74 and δ_C_ 150.4) as an internal standard. ESI-FTMS analyses were measured on a Bruker Magnex BioAPEX 30es ion cyclotron HR HPLC-FT spectrometer by direct injection into an electrospray interface. HPLC purifications were carried out on a Waters 2695 model system equipped with dual absorbance UV detector.

### 3.2. Tunicate Material

The tunicate (ID: KY10508001) was collected from Little Cayman Island at a depth of 10 m. A voucher specimen of the sample was deposited at the NOAA Ocean Biotechnology Center and Repository, Oxford, MS, USA.

### 3.3. Extraction and Isolation

Exactly 53.4 g of freeze-dried and finely ground tunicate material was exhaustively extracted with CH_2_Cl_2_/MeOH (1:1) to yield 5.5 g of the crude extract after concentration under reduced pressure. The crude extract was subjected to vacuum liquid chromatography on C_18_ column using gradient (100:0 to 0:100 H_2_O/MeOH). The 75% MeOH in water fraction (fraction D, 678.7 mg) which showed anti-inflammatory activity was further subjected to repeated reverse phased semi-preparative HPLC purification on Phenomenex, Luna, C_18_(2), 10 µL, 250 × 21.2 mm; flow rate, 10 mL/min, using a gradient of 40:60 (H_2_O:MeOH with 0.05% TFA) to 100% MeOH in 65 min. Peak 2 was identified as **3** (8.3 mg), peak 3 as **2** (4.5 mg), peak 4 as **1** ( 5.6 mg) and peak 10 as **4** (6.5 mg).

Compound **1**: Colorless solid; 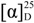: −99.3 (*c*, 0.35, CHCl_3_); UV (MeOH) λ_max_ (log ε) 208 (2.45) 232 (2.63); ^1^H- and ^13^C-NMR data, see Table 1; HRESIMS *m/z* [M + H]^+^ 977.53505/979.53655 (actual 977.53662) (calcd for C_49_H_78_ClN_6_O_12_^+^ 977.53662).

Compound **2**: Colorless solid: 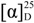: −86.3 (*c*, 0.40, CHCl_3_); UV (MeOH) λ_max_ (log ε) 206 (2.39) 230 (2.49); ^1^H- and ^13^C-NMR data, see Table 1; HERSIMS at *m/z* [M + H]^+^ 963.52073/965.52101 (calcd for C_48_H_77_ ClN_6_O_12_^+^ 963.52097).

Compound **3**: Colorless solid; 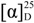: −89.8 (*c*, 0.55, CHCl_3_); HRESIMS *m/z* 943.5461 [M + H]^+^ (calcd for C_49_H_79_N_6_O_12_^+^ 943.5755); ^1^H- and ^13^C-NMR data, see [12,13,14,15].

Compound **4**: Colorless solid; 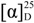: −77.6 (*c*, 0.35, CHCl_3_); HRESIMS *m/z* 1112.6458 [M + H]^+^ (calcd for C_57_H_90_N7O_15_^+^ 1112.6494); ^1^H- and ^13^C-NMR data, see [12,13,14,15].

### 3.4. Biological Assays

Inhibition of NF-κB mediated transcription was determined in SW1353 cells by a reporter gene assay as described by Ma *et al*. [21]. Inhibition of iNOS activity was determined in RAW264.7 cells as described earlier [22]. *In vitro* cytotoxicity was determined against a panel of four solid tumor cell lines and compared with noncancerous kidney cells (Vero) [23]. Parthenolide and doxorubicin were used as positive controls for anti-inflammatory and cytotoxic activity, respectively.

## 4. Conclusions

The investigation of the tunicate *Trididemnum solidum* for bioactive natural products resulted in the isolation of two new chlorinated didemnin classes of compounds in addition to two known compounds. The recent reports of didemnin B from the *α*-proteobacterium *Tistrella mobilis* by Tsukimoto *et al.* [13], didemnin B and nordidemnin B from several species of *Tistrella* by Xu *et al.* [24] suggests that the chlorinated derivatives reported in this paper could also be of bacterial origin. Compound (**1**–**4**) exhibited significant inhibition of NF-κB and iNOS activity, as well as strong anticancer activity towards four cancer cell lines. Compound **1**, the chlorinated derivative was less active compared to **3** in anti-inflammatory and cytotoxicity assays.

## Supplementary Files

Supplementary File 1 Supplementary Materials (PDF, 376 KB)
