# Supplementary material for: Chlorinated Didemnins from the Tunicate Trididemnum solidum
Source: Mar Drugs. 2013 Nov 11;11(11):4478–86. doi: 10.3390/md11114478 (PMC3853740; doi:10.3390/md11114478)

Supplementary Materials

Figure S1. ^{13}C NMR spectrum of compound **1**.

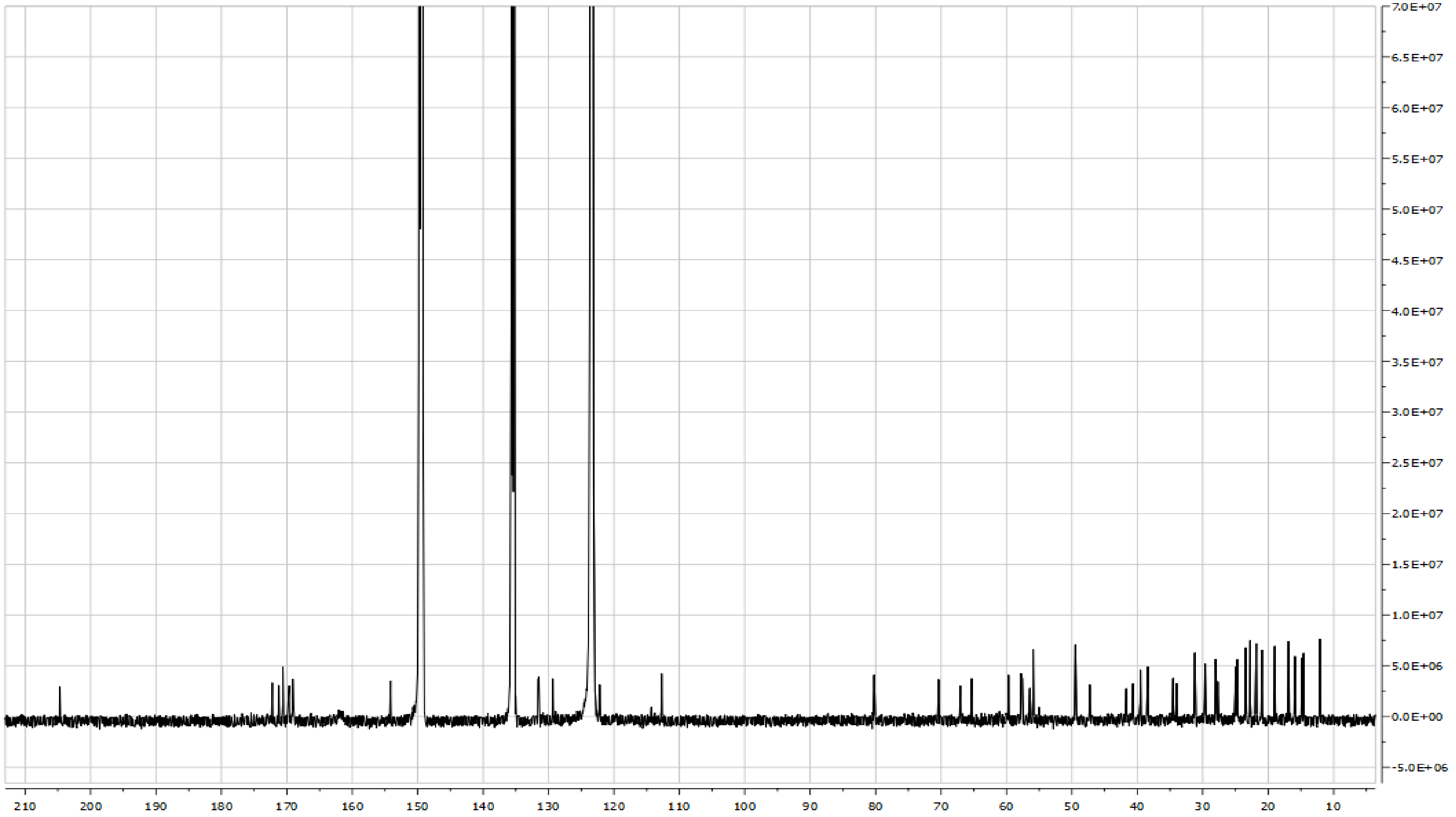

Figure S2. ^{1}H NMR spectrum of compound **1**.

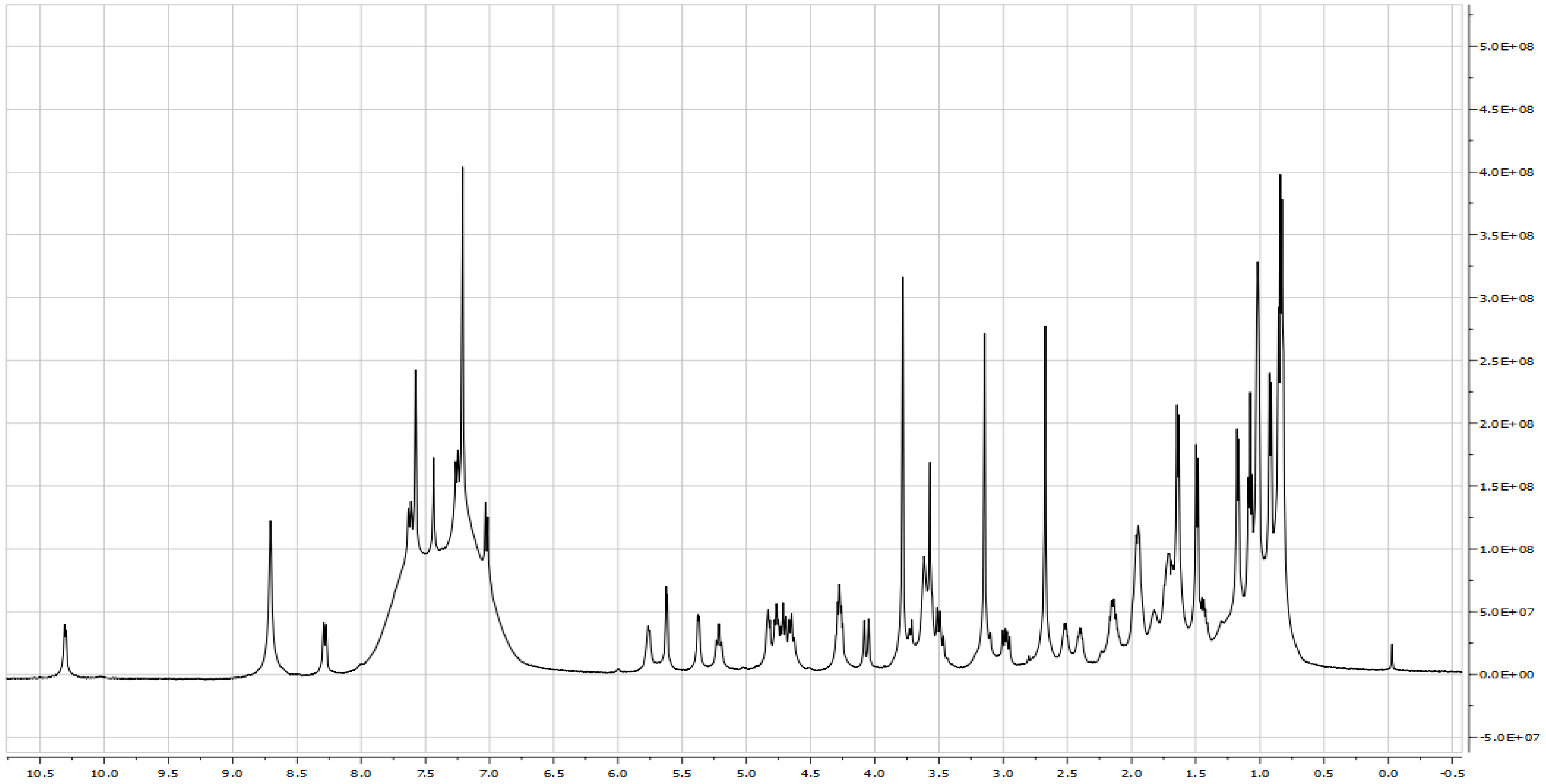

Figure S3. Mass spectrum of compound **1**.

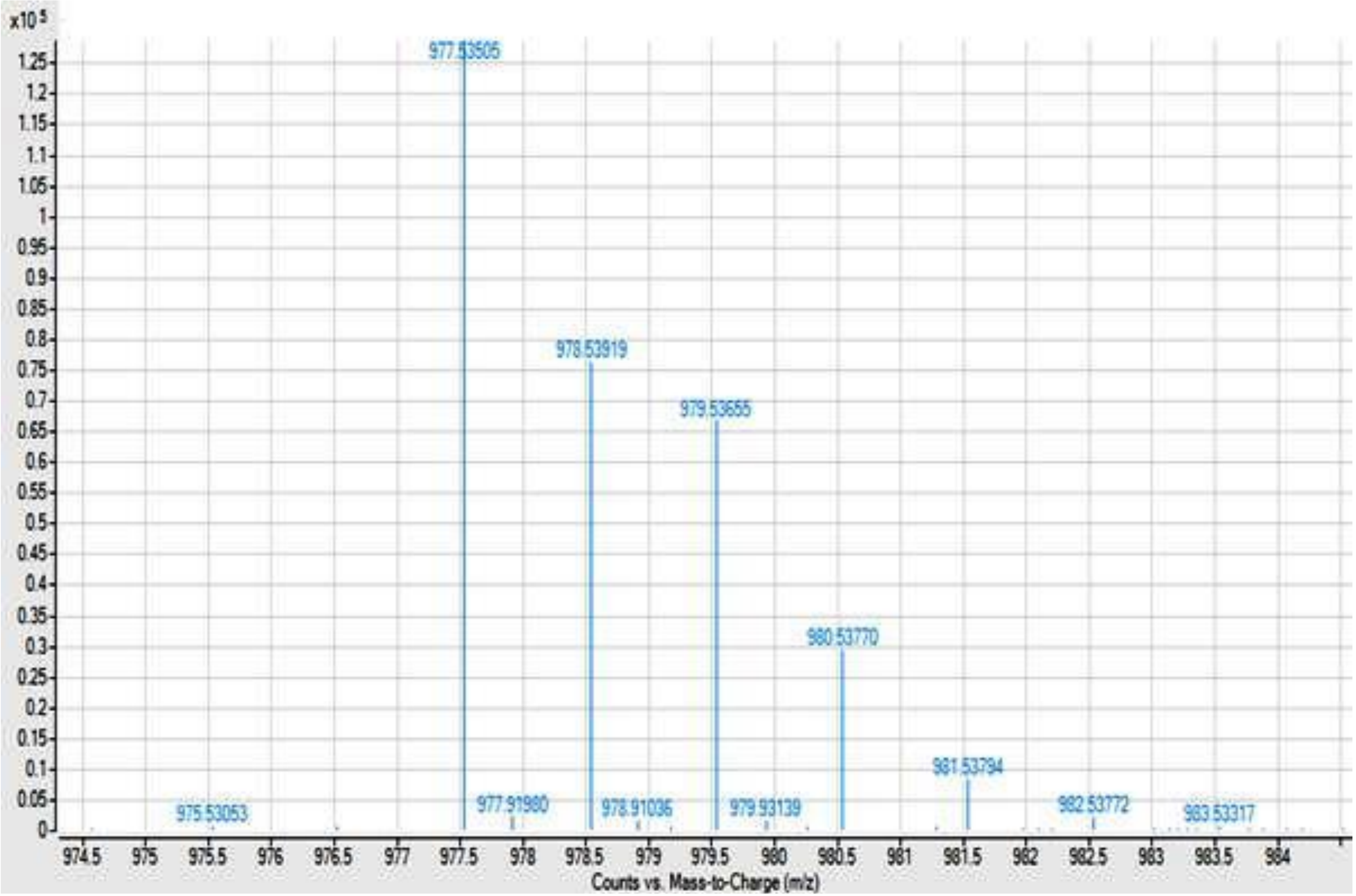

Figure S4. LC-TOF/MS chromatogram and MS spectrum of Marfey's derivatives of compound **1**.

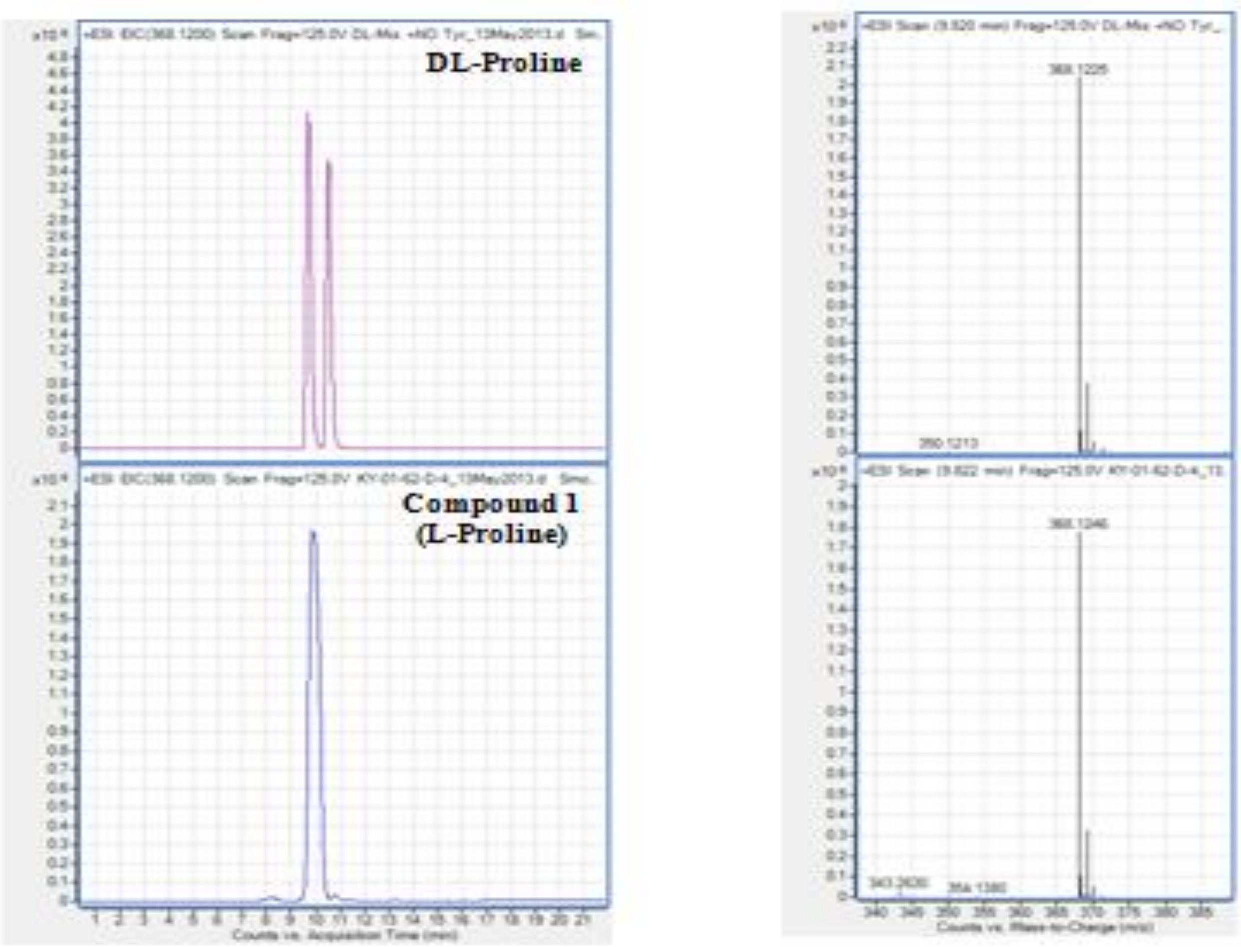

Figure S4. *Cont.*

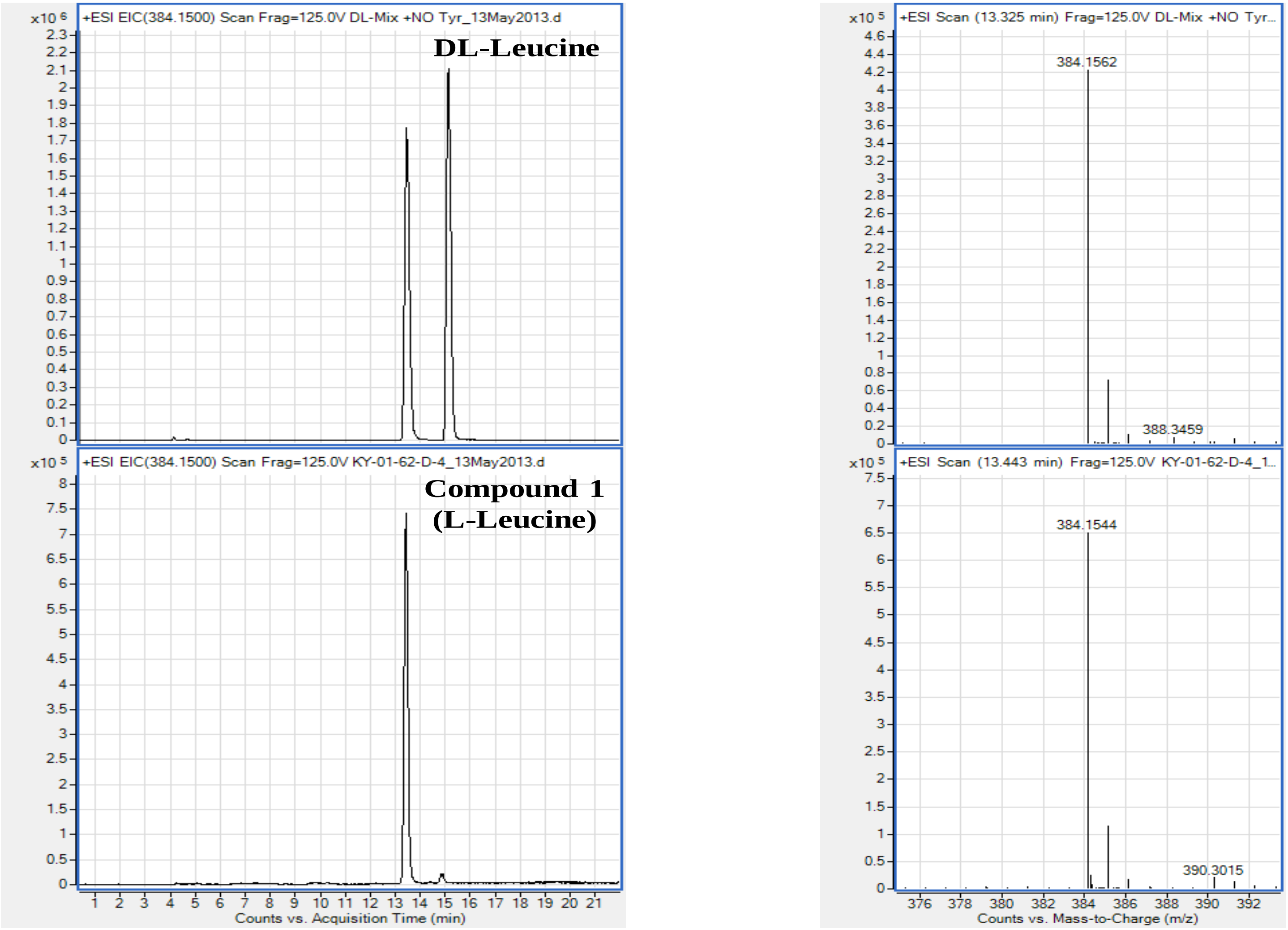

Figure S4. *Cont.*

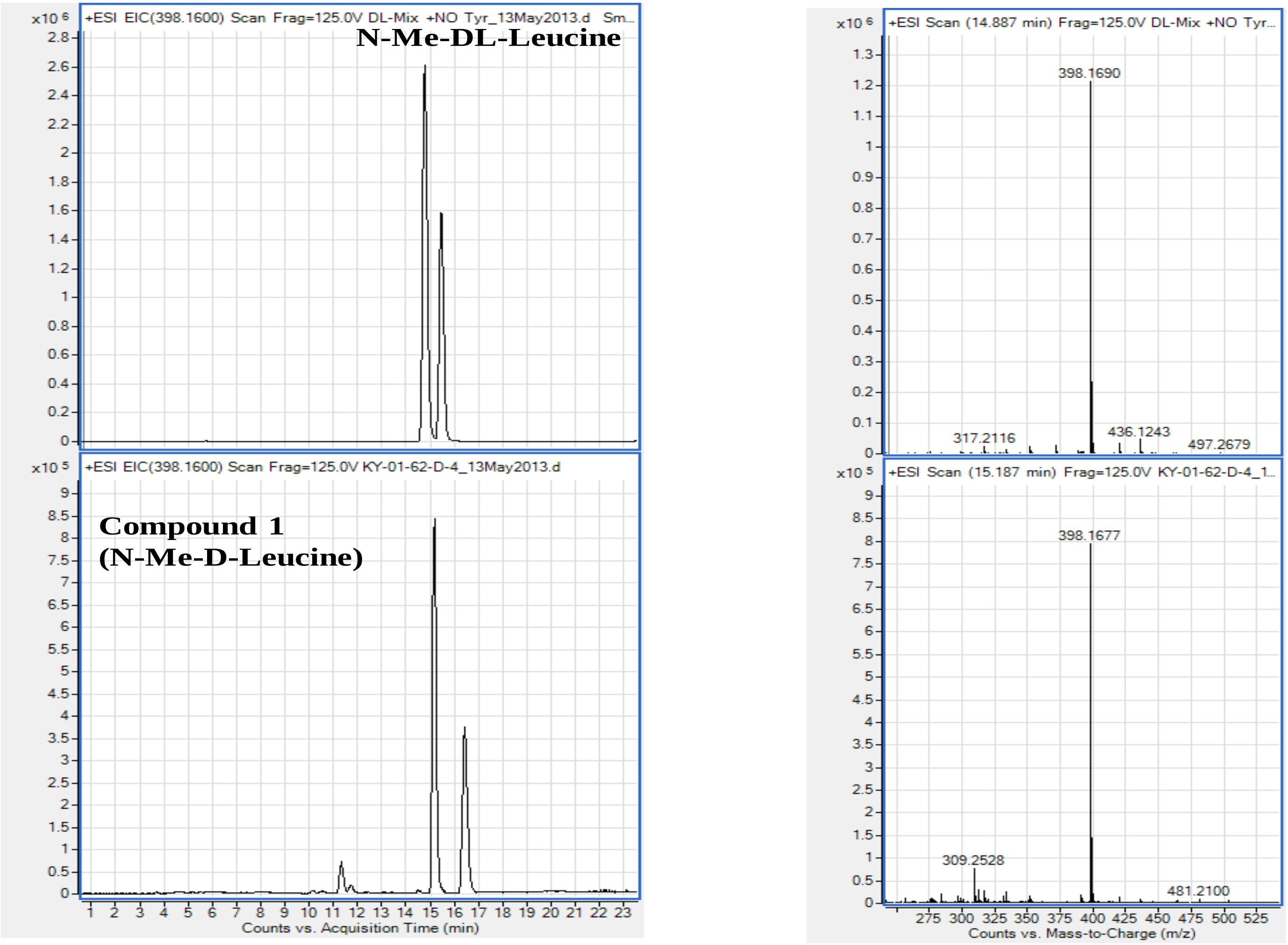

Figure S4. *Cont.*

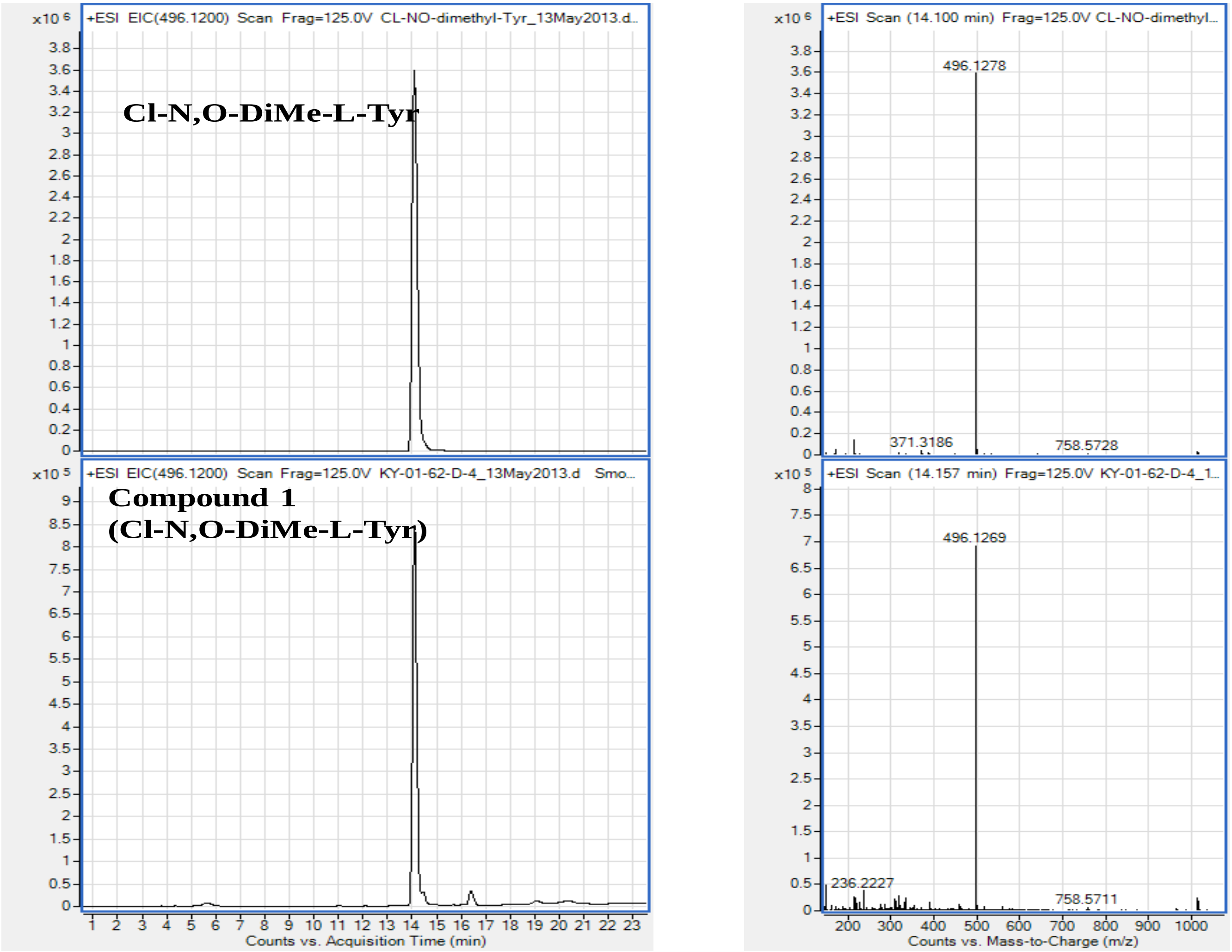

Figure S5. Mass spectrum of compound **2**.

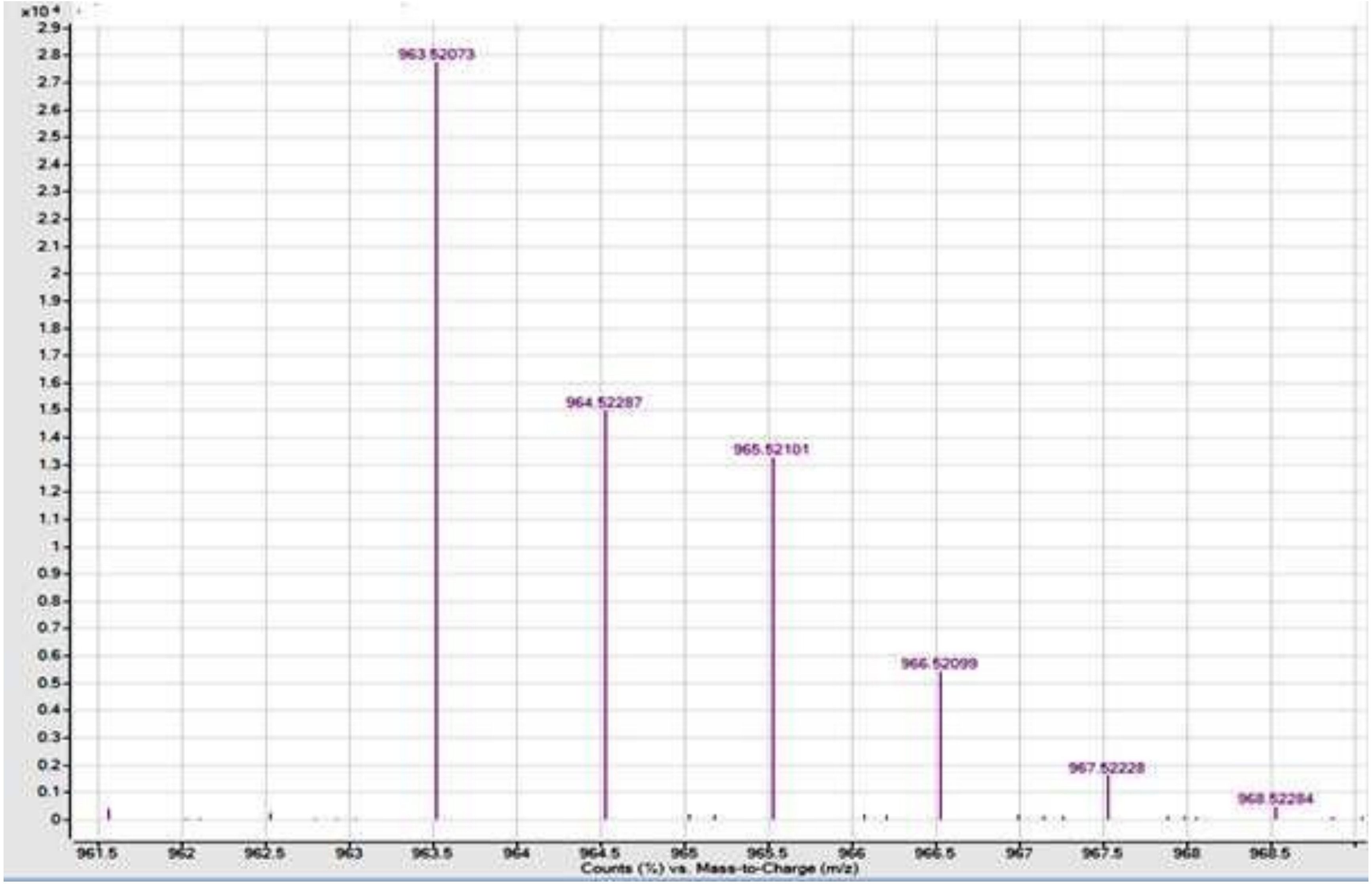

Supplement: Supplementary File 1 — Supplementary Materials (PDF, 376 KB) [file marinedrugs-11-04478-s001.pdf]
